# Comparison of the Screening Tests for Gestational Diabetes Mellitus between “One-Step” and “Two-Step” Methods among Thai Pregnant Women

**DOI:** 10.1155/2018/1521794

**Published:** 2018-02-08

**Authors:** Suchaya Luewan, Phenphan Bootchaingam, Theera Tongsong

**Affiliations:** Department of Obstetrics and Gynecology, Faculty of Medicine, Chiang Mai University, Chiang Mai, Thailand

## Abstract

**Objective:**

To compare the prevalence and pregnancy outcomes of GDM between those screened by the “one-step” (75 gm GTT) and “two-step” (100 gm GTT) methods.

**Methods:**

A prospective study was conducted on singleton pregnancies at low or average risk of GDM. All were screened between 24 and 28 weeks, using the one-step or two-step method based on patients' preference. The primary outcome was prevalence of GDM, and secondary outcomes included birthweight, gestational age, rates of preterm birth, small/large-for-gestational age, low Apgar scores, cesarean section, and pregnancy-induced hypertension.

**Results:**

A total of 648 women were screened: 278 in the one-step group and 370 in the two-step group. The prevalence of GDM was significantly higher in the one-step group; 32.0% versus 10.3%. Baseline characteristics and pregnancy outcomes in both groups were comparable. However, mean birthweight was significantly higher among pregnancies with GDM diagnosed by the two-step approach (3204 ± 555 versus 3009 ± 666 g; *p*=0.022). Likewise, the rate of large-for-date tended to be higher in the two-step group, but was not significant.

**Conclusion:**

The one-step approach is associated with very high prevalence of GDM among Thai population, without clear evidence of better outcomes. Thus, this approach may not be appropriate for screening in a busy antenatal care clinic like our setting or other centers in developing countries.

## 1. Introduction

Gestational diabetes mellitus (GDM) is defined as any degree of glucose intolerance with onset or first recognition during pregnancy, excluding overt diabetes in early pregnancy [1]. GDM causes complications in both mother and baby such as macrosomia, shoulder dystocia, birth trauma, increased rate of cesarean section, death fetus in utero, neonatal hypoglycemia, congenital anomalies, and respiratory distress syndrome [2]. Additionally, women with GDM have a higher rate of postpartum diabetes [2]. Screening for GDM and its early detection and treatment can prevent and reduce harm to both the mother and the baby.

Current screening for GDM in our practice was based on recommendation by American College of Obstetricians and Gynecologists (ACOG) [2] with the two-step approach, screening the women at high and moderate risk. A 50 g glucose challenge test (50 g GCT) is done, followed by a 100 g oral glucose tolerance test (100 g OGTT) in case of a positive screen (>140 mg/dL). In 2010, the International Association of Diabetes and Pregnancy Study Groups (IADPSG), an international consensus group with representatives from multiple obstetrical and diabetes organizations, has proposed guidelines relied on a study of Hyperglycemia and Adverse Pregnancy Outcomes (HAPO) [3]. This new strategy is based on the one-step approach by omitting the 50 g GCT and simplifying diagnostic testing by performing a 75 gram two-hour OGTT and requiring only a single elevated value for diagnosis rather than the previous three-hour OGTT requiring two elevated values for diagnosis. The ADA endorsed and reaffirmed this one-step approach proposed by the IADPSG in 2013 [4]. This new recommendation is based on demonstrated associations between glycemic levels and an increased risk of obstetric and perinatal morbidities. Nevertheless, this approach has not been endorsed by ACOG [5] or by a 2013 NIH Consensus Conference [6]. ACOG emphasizes that 18 percent of all pregnant women will be diagnosed with GDM using the IADPSG criteria due to a lower threshold. They believed that the one-step approach with new criteria would increase health care costs in the absence of solid evidence for improvements in maternal or neonatal outcomes [5]. Most recently, ADA guidelines now leave the option between the one-step IADPSG screening strategy and the two-step screening strategy [1].

Since the new strategy using 75 g OGTT as one-step screening is still controversial, and the prevalence of GDM varies among different racial groups, together with that it has never been tested in our obstetric population or those in most developing countries, where the people are biophysically different, we conducted this study to evaluate its effectiveness in our population. The primary objective of this study was to compare the prevalence of GDM diagnosed by the one-step method or 75 g OGTT using the IADPSG criteria and that diagnosed by the two-step method or ACOG approach, among pregnant Thai women with low and average risk. The secondary objective was to compare the pregnancy outcomes between the two groups.

## 2. Patients and Methods

A prospective descriptive study was conducted at Maharaj Nakorn Chiang Mai Hospital, Chiang Mai University, Thailand, between January 1, 2012 and December 31, 2013, with ethical approval by the institute's review board. Pregnant women attending the antenatal care clinic (ANC) were counseled and invited to join the study after written informed consent was given. Inclusion criteria consisted of (1) singleton pregnancy with low or average risk for GDM, (2) maternal age of 18 years or more and, and (3) gestational age between 24 and 28 weeks of gestation, based on regular menstrual period and ultrasound examination in the first half of pregnancy. Exclusion criteria were pregnant women with high risk for GDM, including known cases of pregestational diabetes or high risk for gestational diabetes mellitus such as previous child birthweight of more than 4,000 grams, previous diagnosis of GDM, obesity or BMI or 30 kg/m^2^ or more, previous neonatal hypoglycemia, and first degree relatives with diagnosed DM or glucosuria. The low or average risk for GDM was defined by any pregnancies that had no criteria for the high risk as mentioned above. The women were offered to have the one-step or two-step method based on their preference.

One-step screening was based on 75 gram two-hour oral glucose tolerance test (75 g OGTT) with at least one abnormal result: fasting plasma glucose ≥92 mg/dL (5.1 mmol/L), but <126 mg/dL (7.0 mmol/L) or one-hour OGTT ≥180 mg/dL (10.0 mmol/L) or two-hour OGTT ≥153 mg/dL (8.5 mmol/L). The two-step approach was as follows: firstly, a 50 g oral glucose challenge test (GCT) was performed regardless of the fasting status. If the plasma glucose level after 1 h was of ≤140 mg/dL, it was considered as negative and needed no further test. If the level was >140 mg/dL, then 100 g OGTT was performed. The plasma glucose was measured after 100 g load at fasting, 1-, 2-, and 3-hour interval. The cut-off values were as follows: fasting ≥105 mg/dL, 1 h ≥ 188 mg/dL, 2 h ≥ 165 mg/dL, and 3 h ≥ 140 mg/dL. A diagnosis of GDM was made if at least two values exceeded the cut-off.

If GDM was diagnosed, the patients were taken care as a standard guideline for diabetic patients during pregnancy. Blood glucose was usually controlled with diabetic diet and exercise. Insulin was used only when fasting glucose was more than 105 mg/dL. The target was to maintain fasting glucose <95 mg/dL or 2-hour postprandial glucose <120 mg/dL. In cases of normal results, routine standard antenatal care was instituted. All of the recruited women were followed up by the authors until delivery.

The main outcome was the prevalence of GDM, and the secondary outcomes included birthweight, gestational age at delivery, rates of preterm birth (delivery before completed 37 weeks), small-for-gestational age (birthweight of less than 5th percentile for each gestational week), large-for-gestational age (birthweight of greater than 90th percentile for each gestational week), low Apgar scores (less than 7 at 5 minutes), cesarean section, and pregnancy-induced hypertension.

### 2.1. Statistical Analysis

Recorded data were analyzed using computers and software packages, using IBM SPSS version 21.0 (IBM SPSS Statistics for Windows, released 2012. Armonk, NY:  IBM Corp). Baseline characteristics and pregnancy outcomes between the two methods were compared, using Student's *t*-test for continuous data and chi-square for categorical data. The normality of the distribution was tested using Kolmogorov-Smirnov test and Shapiro-Wilk test. Statistical significance was set to 0.05.

## 3. Results

A total of 715 women were screened, and 648 were completely followed up with available final outcomes: 278 in the one-step group and 370 in the two-step group. All were Thai ethnics. The prevalence of GDM was significantly higher in the one-step group than that in the two-step group: 89/278 (32.0%) versus 38/370 (10.3%), respectively. No any case of overt DM was identified when defined as fasting hyperglycemia of higher than 126 mg/dL. However, five women had fasting glucose of ≥105 mg/dL and needed insulin. Baseline characteristics, including maternal age, maternal weight, BMI, gestational age at screening, and parity, were not significantly different between the two groups with GDM and the normal group as shown in Table 1. Pregnancy outcomes, including birthweight, gestational age, rates of preterm birth, small-for-gestational age, macrosomia, low Apgar scores (less than 7 at 5 minutes), cesarean section, and pregnancy-induced hypertension, were all not significantly different between the two groups as shown in Table 2.

In the subgroup analysis, considering only pregnancies diagnosed of GDM by the two methods, the baseline characteristics and pregnancy outcomes were comparable in both groups as presented in Tables 3 and 4. However, mean birthweight was significantly higher among pregnancies with GDM diagnosed by the two-step method when compared to that by the one-step method (3204 ± 555 versus 3009 ± 666 g; *p*=0.022). Likewise, the rate of large-for-date had a tendency to be higher in the two-step group, but was not significant.

Comparing the women with GDM and non-GDM, diagnosed by either methods, birthweight among the women with GDM was significantly higher than that of women with non-GDM (3127 ± 658 versus 2932 ± 517; *p* < 0.001), and gestational age of the women with GDM was lower but not statistically significant (37.8 ± 2.9 versus 38.1 ± 2.4; *p*=0.225). The rate of preterm birth also was significantly higher among women with GDM (15.5% versus 8.8%, *p*=0.021).

## 4. Discussion

The 75 gram two-hour OGTT is recommended by the IADPSG and ADA because it is more sensitive for identifying the pregnancy at risk for adverse outcome than the approach, 100 gram three-hour OGTT, recommended by ACOG. Increased sensitivity is likely related to a lower threshold for a positive test; only one elevated glucose value is needed, and the cut-offs are slightly lower. Nevertheless, cost-benefit analysis of the new one-step approach used among other different groups of populations is needed to be explored, and it is directly associated with the prevalence of GDM which is varied with ethnic groups and diagnostic techniques as seen in our study. The prevalence derived by the one-step method was three times greater than the two-step method.

GDM is commonly diagnosed in our practice using the two-step approach; a 1-hour screening test with a 50-gram glucose challenge followed by a 3-hour 100 gram glucose tolerance test in cases of abnormal screen, identifying approximately 5–10% of our population as seen in most reports [6]. Obviously, the prevalence of 32% in pregnant women with low to average risk in our study, as a result of 75 g OGTT screening, was unacceptably high. Therefore, our findings did not support new guideline of screening with one step using 75 g OGTT. The main problem is its too high sensitivity and presumably high false positive rate. This preliminary study suggested that the 75 g OGTT may not be appropriate for routine screening in a busy antenatal care clinic like our setting or other health care centers in developing countries, since the screening test is relatively complicated, involving fasting, without strong evidence of obvious clinical benefit in term of pregnancy outcomes as shown in this study. From our point of view, to adopt the new guideline in other populations, further studies in cost-effectiveness must be thoroughly evaluated, similarly as suggested by ACOG [2].

The very high prevalence in this study may partly be associated with a too high sensitivity of 75 g OGTT. This approach differs on whether a 1-hour sample is included, whether two abnormal values are required, and the diagnostic cut-offs that are used, resulting in high sensitivity. However, racial factor may also be implicated since the prevalence of GDM varies worldwide and among racial and ethnic groups [7–10]. For example, in USA, prevalence rates are higher in African American, Hispanic American, and Asian women than in Caucasian [11]. Moreover, the prevalence has been increasing over time, possibly associated with an increase in mean maternal age as well as maternal weight [12, 13]. It may be reasonable to assume that the high prevalence in our study was likely to be related to the threshold cut-off of the diagnostic test and probably a racial factor. Therefore, prior to implementation in each racial group, this new strategy suggested by IADPSG and ADA should be tested in their own population.

The one-step approach holds potential advantages for women and their health care providers as it would allow a diagnosis to be achieved within the context of one visit instead of two. However, the increased prevalence mentioned above raises several concerns regarding the women with additionally diagnosed GDM. It is not clear whether the additional women detected by the new approach will benefit from treatment, and if so, to what extent. Additionally, the care of these women will certainly increase much health care costs. Moreover, the women labeled for GDM may have unintended consequences, such as an increase in rates of cesarean section and more intensive newborn assessments, increased patient costs, and possible psychosocial burdens.

Note that, among pregnancies diagnosed with GDM, birthweight was significantly higher when diagnosed by the two-step method, indicating that the one-step method was possibly more sensitive and a large number of nonlarge fetuses could be more recruited. However, birthweight could be affected by several factors, especially the effect of GDM treatment.

The following are the weaknesses of this study: (1) small sample size for some secondary outcomes, especially a rare event like perinatal death; (2) no randomization for allocation of the patients to each group of the study; however, this might have only minor effect on the comparison since the baseline characteristics of the women were similar; and (3) therapeutic effect may probably obscure the pregnancy outcomes. For example, diet control in women with GDM could modify pregnancy outcomes like birthweight or rate of large-for-date. Certainly, this therapeutic confounder could not be eliminated due to ethical reason. Finally, due to no screening for overt DM in early gestation, unidentified pregestational diabetes could be included in the recruited women; however, this could only minimally affect our conclusion since only very few cases of overt DM was detected in this study.

In conclusion, this study concerned that the adoption of the one-step method would markedly increase the prevalence of GDM, three times greater than the two-step method, and the relevant costs and interventions, without clear demonstration of improvements in the most clinically important health and patient-centered outcomes. This study provides important clinical data to encourage physicians to explore the most appropriate screening strategy for their own population. Probably, this new approach may not be appropriate for screening in a busy antenatal care clinic like our setting or other health care centers in developing countries.

## Figures and Tables

**Table 1:** Comparison of baseline characteristics between the one-step and two-step groups.

Characteristics	One step (*n* = 278)	Two step (*n* = 370)	*p* value
Maternal age	27.9 ± 5.6	27.8 ± 5.4	0.795
Maternal weight (at screening)	60.4 ± 8.7	60.5 ± 8.5	0.993
Maternal BMI	21.5 ± 5.8	21.6 ± 6.8	0.756
Prepregnancy weight	51.9 ± 7.6	51.8 ± 7.5	0.854
Gestational age at screening (weeks)	25.8 ± 1.4	25.8 ± 1.4	0.856
Fasting blood glucose	84 ± 7	84 ± 7	0.912
Multiparity, *n* (%)	120 (43.2%)	159 (43.0%)	0.512

**Table 2:** Comparison of pregnancy outcomes between the one-step and two-step groups.

Outcomes	One step (*n* = 278)	Two step (*n* = 370)	*p* value
Gestational age at delivery (weeks)	38.4 ± 2.3	38.1 ± 2.2	0.882
Birthweight (grams)	2981 ± 551	2962 ± 556	0.645
Neonatal glucose	42 ± 37	41 ± 36	0.912
Preterm birth	26 (9.4%)	41 (11.1%)	0.474
Low Apgar score, *n* (%)	11 (4.0%)	24 (6.5%)	0.159
Small-for-gestational age (<5 centile), *n* (%)	10 (3.6%)	12 (23.2%)	0.341
Large-for-gestational age, *n* (%)	32 (11.5%)	40 (10.8%)	0.828
Pregnancy-induced hypertension, *n* (%)	21 (7.6%)	31 (8.4%)	0.409
Cesarean section rate, *n* (%)	59 (21.2%)	77 (20.8%)	0.809

**Table 3:** Comparison of baseline characteristics of pregnancies with GDM between the groups diagnosed by the one-step and two-step techniques.

Characteristics	One step (*n* = 89)	Two step (*n* = 38)	*p* value
Maternal age	28.7 ± 5.0	28.5 ± 5.3	0.903
Maternal weight (at screening)	60.4 ± 10.3	61.3 ± 7.6	0.617
Maternal BMI	22.2 ± 9.5	21.5 ± 2.8	0.664
Prepregnancy weight	52.2 ± 8.0	52.3 ± 8.2	0.974
Gestational age at screening (weeks)	25.5 ± 1.4	25.6 ± 1.5	0.788
Fasting blood glucose	88 ± 7	88 ± 5	0.879
Multiparity, *n* (%)	36 (40.4%)	20 (52.6%)	0.205

**Table 4:** Comparison of pregnancy outcomes of pregnancies with GDM between the groups diagnosed by the one-step and two-step techniques.

Outcomes	One step (*n* = 89)	Two step (*n* = 38)	*p* value
Gestational age at delivery (weeks)	37.5 ± 3.1	38.5 ± 2.2	0.083
Birthweight (grams)	3009 ± 666	3204 ± 555	0.022
Neonatal glucose	55 ± 34	64 ± 28	0.080
Preterm birth	15 (16.9%)	6 (15.8%)	0.882
Low Apgar score, *n* (%)	1 (1.1%)	0 (0.0%)	0.512
Small-for-gestational age (<5 centile), *n* (%)	5 (5.6%)	2 (5.3%)	0.841
Large-for-gestational age, *n* (%)	12 (13.5%)	11 (28.9%)	0.115
Pregnancy-induced hypertension, *n* (%)	6 (6.7%)	3 (7.9%)	0.817
Cesarean section rate, *n* (%)	20 (22.5%)	10 (26.3)	0.641
